# Relationship Between Repeated Sprint Performance and both Aerobic and Anaerobic Fitness

**DOI:** 10.2478/hukin-2014-0016

**Published:** 2014-04-09

**Authors:** Wajdi Dardouri, Mohamed Amin Selmi, Radhouane Haj Sassi, Zied Gharbi, Ahmed Rebhi, Mohamed Haj Yahmed, Wassim Moalla

**Affiliations:** 1 Department of physical Education, University of Hail-College of Education, Hail, Kingdom of Saudi Arabia.; 2 Research Unit « School and University Sportive Practices and Performance », High Institute of Sports and Physical Education, Kef, University of Jendouba, Tunisia.; 3 Tunisian Research Laboratory “Sport Performance Optimisation”, National Center of Medicine and Science in Sports (CNMSS), Tunis, Tunisia.; 4 King Abdulaziz University – Faculty of Education in Jeddah. Physical Education Department, Kingdom of Saudi Arabia.; 5 Research Unit, « Analysis and Evaluation of Factors Affecting the Sports Performance », High Institute of Sports and Physical Education, Ksar Said, University of Manouba, Tunisia.; 6 Research Unit: EM2S, High Institute of Sports and Physical Education, Sfax, University of Sfax, Tunisia.

**Keywords:** aerobic fitness, anaerobic power, vertical jump, horizontal jump, repeated sprint ability

## Abstract

The aims of this study were firstly, to examine the relationship between repeated sprint performance indices and anaerobic speed reserve (AnSR), aerobic fitness and anaerobic power and secondly, to identify the best predictors of sprinting ability among these parameters. Twenty nine subjects (age: 22.5 ± 1.6 years, body height: 1.8 ± 0.1 m, body mass: 68.8 ± 8.5 kg, body mass index (BMI): 22.2 ± 2.1 kg•m-2, fat mass: 11.3 ± 2.9 %) participated in this study. All participants performed a 30 m sprint test (T30) from which we calculated the maximal anaerobic speed (MAnS), vertical and horizontal jumps, 20m multi-stage shuttle run test (MSRT) and repeated sprint test (10 × 15 m shuttle run). AnSR was calculated as the difference between MAnS and the maximal speed reached in the MSRT. Blood lactate sampling was performed 3 min after the RSA protocol. There was no significant correlation between repeated sprint indices (total time (TT); peak time (PT), fatigue index (FI)) and both estimated VO2max and vertical jump performance). TT and PT were significantly correlated with T30 (r=0.63, p=0.001 and r=0.62, p=0.001; respectively), horizontal jump performance (r = −0.47, p = 0.001 and r = −0.49, p = 0.006; respectively) and AnSR (r=−0.68, p= 0.001 and r=−0.70, p=0.001, respectively). Significant correlations were found between blood lactate concentration and TT, PT, and AnSR (r=−0.44, p=0.017; r=−0.43, p=0.018 and r=0.44, p=0.016; respectively). Stepwise multiple regression analyses demonstrated that AnSR was the only significant predictor of the TT and PT, explaining 47% and 50% of the shared variance, respectively. Our findings are of particular interest for coaches and fitness trainers in order to predict repeated sprint performance by using AnSR that can easily identify the respective upper performance limits supported by aerobic and anaerobic power of a player involved in multi-sprint team sports.

## Introduction

In the last decade, it has been widely regarded that repeated sprint ability (RSA) is considered a relevant fitness component in team sports (Buchheit et al., 2010a; Buchheit et al., 2010b; Castagna et al., 2007). In this context, a various range of testing protocols were used to either evaluate RSA (Haj-Sassi et al., 2011; Spencer et al., 2011), or as a tool of training program prescription (Buchheit et al., 2010c). Nevertheless, fitness attributes and energetic requirements of RSA are still a subject of debate among researchers and fitness coaches (Gaitanos et al., 1993; Spencer et al., 2011). Since both PCr replenishment and the removal of accumulated intracellular Pi are oxygen-dependent processes, it was hypothesized that there was a relationship between aerobic fitness and fatigue during multiple sprint exercises (Glaister, 2005). However, the relationship between maximal aerobic power and performance in repeated sprint activities has not always been identified. In fact, although some studies reported significant correlations between VO_2_max and RSA performance indices (Bishop and Edge, 2006; Bishop and Spencer, 2004; Glaister, 2007; Glaister, 2005), others have failed to do so (Aziz et al., 2007; Bishop et al., 2003; Castagna et al., 2007). Moreover, the ability to perform repeated sprints was also closely related to anaerobic attributes such as anaerobic glycolysis (Glaister, 2008), the ability to buffer hydrogen ions (Bishop et al., 2004; Edge et al., 2006) and muscle glycogen concentration (Gaitanos et al., 1993). Indeed, high concentrations of lactate recorded during repeated sprints of various protocols (>10 mmol/l) suggest that anaerobic glycolysis solicitation is not negligible (Glaister, 2008). It could be assumed therefore, that repeated sprint tests require significant energy contributions from the phosphate, anaerobic glycolysis and aerobic systems. Nevertheless, the relationship between RSA indices and both aerobic and anaerobic indices is still a controversial subject among researchers.

The anaerobic speed reserve model (AnSR), which represents the difference between the maximal anaerobic speed and the maximal aerobic velocity, has been previously proposed to predict performance in running events ranging from 3 s to 240 s (Blondel et al., 2001; Bundle et al., 2003; Weyand and Bundle, 2005). It has been also suggested that AnSR could be used as a performance assessment alternative to existing tests of anaerobic power and capacity. Additionally, Blondel et al. (2001) suggested that expressing supra-maximal velocity as a percentage of AnSR allows individual differences in anaerobic work capacity to be taken into account and running times to exhaustion to be predicted accurately. Recently, Mendez Villanueva et al. (2008) reported a significant correlation (r = 0.87, p ≤ 0.05) between the anaerobic power reserve and the fatigue index during 10 × 6 s sprints on a cycle ergometer. These authors speculated that subjects with low anaerobic power reserves, implying less dependence on anaerobic metabolism, showed a higher resistance to fatigue during repeated bouts of supra-maximal cycling. However, subjects with higher anaerobic power reserves, implying a greater reliance to anaerobic energetic systems, recorded a larger power decrement during the ten cycling sprints. Nevertheless, this study was carried out on a very small population (8 subjects) and used cycling exercise, and not a sport-specific test setting, which limits its transfer to running exercises. On the other hand, as suggested by Bishop et al. (2011), training interventions that target the main factors limiting RSA may be a more effective approach to improve repeated sprint performance.

In order to better serve coaches and professionals of athletic training in the identification of variables that can effectively improve performance in repeated sprint tests, the purposes of our study were as follows: 1) to examine the relationship between the repeated sprint performance indices and anaerobic speed reserve (AnSR), aerobic fitness (estimated VO_2_max) and anaerobic power (straight sprint, vertical and horizontal jump tests) and 2) to identify the best predictors of repeated sprint ability among these variables.

## Material and Methods

### Participants

Twenty nine male sport science students (age: 22.5 ± 1.6 years, body height: 1.8 ± 0.1 m, body mass: 68.8 ± 8.5 kg, body mass index (BMI): 22.2 ± 2.1 kg·m^−2^, fat mass: 11.3 ± 2.9 %) participated in this study. They were licensed in various team sports (football, basketball, rugby and handball). Subjects were selected based on their team-sport experience (each subject had at least 5 years of training experience). All subjects trained regularly (6 ± 2 sessions per week) in addition to one competition per week. None of the participants reported any current or ongoing neuromuscular diseases or musculoskeletal injuries specific to the ankle, knee, or hip joints, and none of them were taking any dietary or performance enhancing supplements that could affect results of the study. The protocol prepared according to the Declaration of Helsinki 1975 was fully approved by the local Ethical Committee of High Institute of Sports and Physical Education, Kef, University of Jendouba, Tunisia, and a written informed consent form was obtained from all subjects after explanation of the experimental design and potential risks of the study.

### Procedure

All testing sessions were conducted indoor. The exercise sessions were separated by at least one week. During the first session the subjects performed a 30 m sprint test, as well as vertical and horizontal jumps. All subjects performed 2 trials for each test with at least 3 min of rest between trials and 5 min between tests to ensure adequate recovery. The better performance of the 2 trials was used for further statistical analysis. During the second session, the subjects performed the 20 m multi-stage shuttle run test (MSRT) to estimate VO_2_max through the maximal speed reached during the test. During the last session, the participants performed the repeated sprint ability test (RSA). Except for MSRT, all other tests were preceded by a 15 min warm-up including jogging, dynamic stretching, jumping and accelerations. All tests were performed at the same time of day (±1h) and subjects were asked to follow their normal diet and to refrain from any form of intense physical activity 48 h prior to testing sessions.

### Maximal Anaerobic Speed Test (MAnS)

The subjects performed a maximal sprint over 30 m (T30). The performance of the test was recorded using 3 pairs of photocell gates (Brower timing system, Salt Lake City, UT, USA; accuracy of 0.01s) placed approximately 0.75 m above the floor and positioned 3 m apart facing each other on either side of the starting line, at 10 m and at the finish. Subjects were instructed to begin with their preferred foot forward, placed on a line marked on the floor from a standing position. The time of the 10–30 m phase was used as the criterion measure of MAnS.

### Countermovement Jump (CMJ)

The subjects began from an upright standing position. They performed a very fast preliminary downward eccentric action followed immediately by a vertical jump for maximal height. Hands remained at the hips for the entire movement to eliminate any influence of arm swing. CMJ performance (peak height) was measured using the optojump system (Microgate, SARL; Italy).

### Standing quantiple jump (5-JT)

The 5JT consists of 5 consecutive hops with joined feet position at the start and end of the jumps. From the starting joined feet position, the participant was not allowed to perform any back step with any foot, rather he had to directly jump to the front with a leg of his choice. After the first four hops, i.e. alternating left and right feet for two times each, he had to perform the last hop and finish up the test with feet together. If ever the player fell to the back at the completion of the last hop, the test was performed over. The 5JT performance was measured with a tape from the front edge of the subject’s feet at the starting position to the rear edge of the feet at the landing.

### 20m Multi-Stage Shuttle Run Test (MSRT)

The MSRT was conducted as previously described by Léger and Lambert (1982). This test consisted of shuttle running between two lines, spaced 20 m apart. The initial velocity of the incremental test was set at 8 km·h^−1^ and increased by 0.5 km·h^−1^ every minute. The subjects adjusted their running velocity according to a combination of regular auditory pacing signals provided by a beeper (Best Electronic, France). All subjects were verbally encouraged to exert their maximal effort possible. It has been shown that the MSRT is a reliable and valid indicator of maximal aerobic power (Léger and Lambert, 1982). The subject was required to stop if on two consecutive laps he failed to arrive within 2 m of the finish line. Maximal speed was calculated as the velocity of the last stage fully completed and considered as the speed associated with VO_2_max for the shuttle run test (vVO_2_max).

### Anaerobic Speed Reserve (AnSR)

AnSR was calculated as the difference between the MAnS and the maximal speed reached in the MSRT (vVO_2_max). MAnS and vVO_2_max were empirically determined values that are representative of the body’s functional limits for a-lactic anaerobic and endurance performances (Blondel et al., 2001; Bundle et al., 2003; Weyand and Bundle, 2005).

$$\text{AnSR}\left(\text{m}\cdot {\text{s}}^{-1}\right)=\text{MAnS}\left(\text{m}\cdot {\text{s}}^{-1}\right)-{\text{vVO}}_{2}\text{max}\left(\text{m}\cdot {\text{s}}^{-1}\right)$$

### Repeated Sprint Ability Test (RSA)

The RSA test consisted of 10 repetitions of 30 m shuttle sprints (15 + 15 m) interspersed with 30 s of passive recovery. Each sprint shuttle was performed with one change of direction (180° turns) and was timed using a photocell system (Brower timing system, Salt Lake City, 174 UT, USA; accuracy of 0.01 s) (Castagna et al., 2007). To avoid pacing, participants were strongly encouraged to exert their maximal effort possible during each of the 30 m shuttle sprints. The following variables were derived from the RSA test: peak time (PT): the best time; (b) total time (TT): the sum of all sprint times; (c) fatigue index (FI): the FI was calculated as recommended by Fitzsimons et al. (1993). Three minutes after the RSA test, blood lactate samples were taken from the fingertip (Lactate Pro; Arkray, Tokyo, Japan).

### Statistical Analysis

Data analysis was performed using SPSS software (SPSS, version 17 for Windows. Inc., Chicago, IL, USA). Values were expressed as mean and SD. Pearson’s product–moment correlation coefficients were used to examine correlations between variables. The magnitude of the correlations was also determined using the modified scale by Hopkins et al. (2009): r < 0.1, trivial; 0.1 – 0.3, small; 0.3 – 0.5, moderate; 0.5 – 0.7, large; 0.7 – 0.9, very large; 0.9, nearly perfect; and 1 perfect. A stepwise multiple regression analysis was used to determine the best predictor variables for TT and PT. Statistical significance was set at p ≤ 0.05.

## Results

Mean ± SD of RSA performance indices, lactate concentration, T30, CMJ, 5JT and AnSR are presented in Table 1.

The correlations between the performance indices of the RSA, lactate concentration, T30, CMJ, 5JT and AnSR are illustrated in Table 2. No significant correlations were found between estimated VO_2_max and TT (r = −0.28, p = 0.139), PT (r = −0.27, p = 0.152) and FI (r = −0.15, p = 0.432).

**Figure 1.**
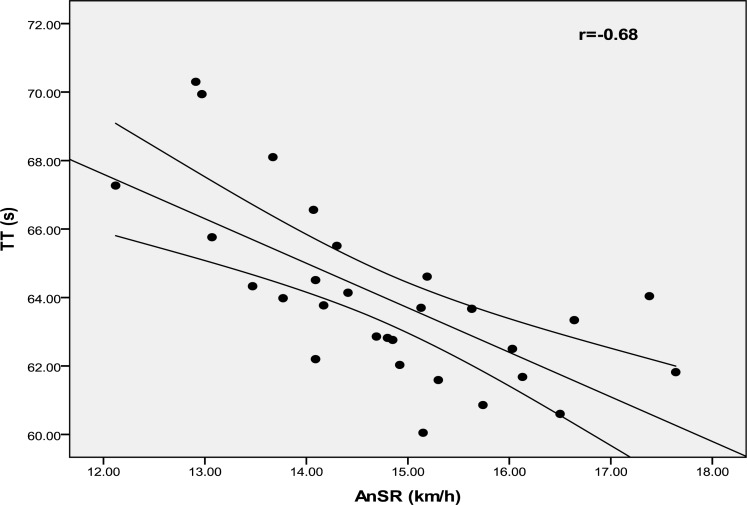
Correlation between total time and anaerobic speed reserve

**Figure 2.**
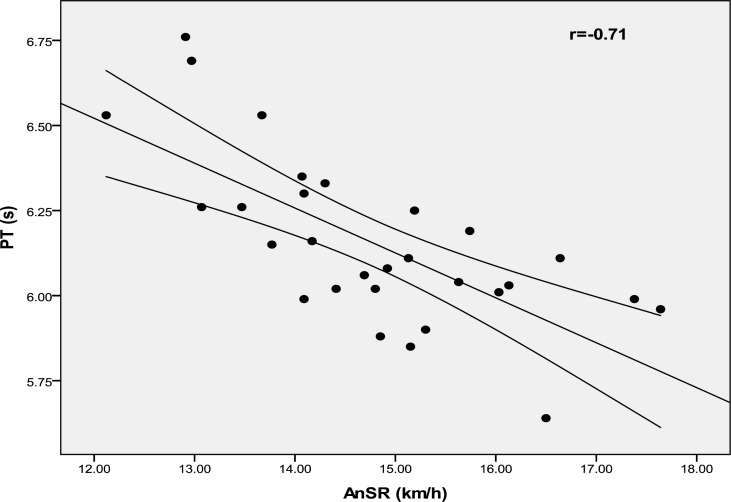
Correlation between peak time and anaerobic speed reserve

Figures 1 and 2 show the relationship between AnSR and both TT and PT. Significant correlations were found between lactate concentration and TT, PT and AnSR (r = −0.44, p = 0.017; r = −0.43, p = 0.018 and r = 0.44, p = 0.016; respectively).

The results of the stepwise multiple regression analyses for RSA are provided in Table 3. AnSR was the only significant predictor of the TT and PT, explaining 47% and 50% of the shared variance, respectively.

## Discussion

The aim of the present study was to examine the relationship between repeated sprint performance indices and aerobic fitness, anaerobic power and AnSR and to identify the best predictors of repeated sprint ability among the different indices used.

Our results highlighted the usefulness of AnSR, in contrast to estimated VO_2_max and other parameters of explosiveness, as the only predictor of repeated sprint performance when expressed as TT and PT. Our data showed no significant relationship between estimated VO_2_max and repeated sprint performance indices (TT, PT and FI). These results corroborate with previous studies (Aziz et al., 2007; Bishop et al., 2003; Castagna et al., 2007). Indeed, Castagna et al. (2007) failed to find significant correlations between VO_2_max and both TT and FI (r = 0.37 and r = −0.28, respectively) in basketball players. Likewise, Bishop et al. (2003) reported a non significant correlation between VO_2_max and FI or other repeated-sprint ability-related variables in a group of female field hockey players. However, other studies have reported significant correlations between VO_2_max and RSA performance indices (Bishop and Edge, 2006; Glaister, 2005). The difference reported by these studies could be due to the different RSA protocols used. In addition, the lack of correlation between VO_2_max and RSA could be due to the fact that maximal aerobic power is thought to be determined essentially by central factors (Bassett and Howley, 2000), while RSA performance is more associated with peripheral factors (Da Silva et al., 2010). Furthermore, VO_2_max is not the only indicator of aerobic fitness. Indeed, aerobic capacity, as represented by anaerobic threshold or the velocity at the onset of blood lactate accumulation (OBLA), could have a greater association with RSA than VO_2_max (Da Silva et al., 2010; Psotta et al., 2011). In fact, Da Silva et al. (2010) showed that repeated sprint indices are more strongly correlated with the OBLA than the more commonly measurement of VO_2_max. Recently, Psotta et al. (2011) reported significant correlations between the average speed of a repeated sprint test and speed corresponding to the ventilatory threshold (r = 0.62, p = 0.01).

The lactate concentration measured after the repeated sprints test was 14.8 ± 0.4 mmol·l^−1^. This result is in agreement with Castagna et al. (2007), who reported lactate concentrations of 14.2 ± 3.5 mmol·l^−1^. This high lactate concentration explains the solicitation of anaerobic glycolysis during the RSA test. Although, Gaitanos et al. (1993) reported a low contribution of anaerobic lactic metabolism during the final sprint of a 10 × 6 s pedaling test, other investigators have observed a gradual increase in lactate concentrations during RSA tests (Glaister, 2005). In the current study, we found a significant correlation between TT and the post exercise lactate concentrations. This finding means that subjects who have achieved the best performance in the repeated sprint test are those who reached higher lactate concentrations, which confirms a major contribution of anaerobic metabolism during repeated sprint tests. This result is confirmed by a significant relationship reported between peak anaerobic power (assessed using the Wingate test) and repeated sprint indices (Bishop et al., 2004; Haj-Sassi et al., 2011).

The AnSR model has been initially proposed to estimate the portion of the power output that can be provided anaerobically (Bundle et al., 2003; Weyand and Bundle, 2005). Our data showed that AnSR was highly correlated with both TT and PT (r = −0.68, p = 0.001, r = −0.70, p = 0.001, respectively). In other words, the higher the AnSR, the better was the TT and PT performed by the subject during the RSA. According to Mendez Villanueva et al. (2008), subjects possessing a high anaerobic power reserve are dependent on anaerobic metabolism during RSA and thereby recorded larger power decrement across the ten cycling sprints. In this regard, Gaitanos et al. (1993) reported that subjects with better sprinting capabilities rely more on anaerobic metabolism to support mechanical function than subjects with lesser ability. This finding is confirmed in our study by the significant correlation between the AnSR and the lactate concentrations after the repeated sprint test (r = 0.44, p < 0.016).

On the other hand, no correlation was found between the repeated sprint performance indices and vertical jump test (CMJ). It has been suggested that vertical jump tests (SJ, CMJ) do not predict performance in repeated sprints with a change of direction (Haj-Sassi et al., 2009). However, Tonnessen et al. (2011) showed a significant moderate correlation between test performance of 10 × 40 m with 60 s recovery and CMJ. It should be noted that our protocol involves a change of direction, which is not the case in the study of Tonnessen et al. (2011). Furthermore, the recovery time between sprints was much higher than that used in our study. It appears that the CMJ as a plyometric test, requiring the stretch shortening cycle muscle in a slow way, does not affect performance of repeated sprints with a change of direction. However, we observed a significant correlation between the 5JT and both TT (r = −0.47, p ≤ 0.01) and PT (r = −0.49, p ≤ 0.006). These results were in agreement with previous research that reported significant correlations between 5JT and both TT and PT (Haj-Sassi et al., 2011; Haj-Sassi et al., 2009). Young et al. (2002) suggested that the change of direction speed depends on the capacity of a subject to generate force in a short period of time with a relatively short ground contact time. It seems that the 5JT, seeking a fast stretch shortening cycle, may influence on repeated sprint performance. Our findings could be of great interest to coaches and fitness trainers to choose plyometric exercises that can effectively improve RSA performance. Future research is needed to test the effect of plyometric training based on horizontal jumps on RSA performance.

The stepwise multiple regression analysis showed that the AnSR was the only significant predictor of the TT and PT, explaining 47% and 50% of the shared variance, respectively. Several studies have attempted to explore the contribution of different energy metabolism during repeated sprints but few of them have examined potential predictors of repeated sprint performance (Glaister, 2005; Spencer et al., 2011). Indeed, Lemmink et al. (2006) showed that VO_2_max and relative peak power developed at the Wingate test explain 58% of TT during the interval shuttle run test. Psotta et al. (2011) also reported that 89% of the performance of 10 × 20 m (expressed in terms of average speed) is explained by the speed of 20 m and the average velocity maintained during an endurance race (2000 m). They also reported 49% of the average speed maintained during the test of repeated sprints was explained by both the running speed corresponding to ventilatory threshold and relative peak power developed in the Wingate test. These results showed that the performance tests of repeated sprints are always predicted from variables derived from the aerobic and anaerobic metabolism. It appeared that AnSR which identify the respective upper performance limits supported by the anaerobic and aerobic power of a subject could predict accurately repeated sprint performance when expressed as TT and PT. Furthermore, it seems that AnSR which incorporates terms directly influenced by the metabolic power available from both anaerobic and aerobic energetic systems could be used to assess performance capabilities during RSA tests.

## Conclusions

In conclusion, our results suggested that anaerobic metabolism contributes mainly to the energy supply during the protocol of RSA (10 × 30 m shuttle run). In addition, we showed that a repeated change of direction sprint performance was related, for the most part, to AnSR. Because tests of aerobic power and single sprint events are commonly used in the routine assessment of multi-sprint team players, our results demonstrated that a simple calculation of AnSR could considerably help coaches and fitness trainers to predict, in a satisfactory way, the performance of RSA in moderately trained men. Further studies are needed to examine the best approach to apply this setting with the aim to improve RSA performance.

## Figures and Tables

**Table 1:** Descriptive data of RSA performance indices, estimated VO_2_max, straight sprint, jump tests, AnSR and lactate concentration after RSA test

Variables	Mean	SD
TT (s)	63.90	2.50
PT (s)	6.15	0.25
FI (%)	4.12	1.40
Estimated VO_2_max (ml/kg/min)	51.84	3.10
T30 (s)	4.49	0.49
CMJ (cm)	37.00	5.10
5JT (m)	13.20	0.52
AnSR (km·h^−1^)	11.50	1.07
[LA (3′)] (mmol·L^−1^	14.78	0.42

TT= total time; PT= peak time; FI= fatigue index; RSA= Repeated sprint ability test; (T30) = 30 m straight sprint test; CMJ= Countermovement Jump, 5JT= Syanding quantiple jump; AnSR= Anaerobic speed reserve; [LA (3′)] = lactate concentration after RSA test.

**Table 2:** Correlation coefficients between RSA indices and test performances and AnSR

	[LA (3′)] (mmol·L^−1^)	T30 (s)	CMJ (cm)	5JT (m)	AnSR (km·h^−1^)
TT (s)	−0.44 (p=0.017)	0.63 (p=0.000)	−0.29 (p=0.132)	−0.47 (p=0.010)	−0.68 (p=0.000)
PT (s)	−0.43 (p=0.018)	0.62 (p=0.000)	−0.30 (p=0.111)	−0.49 (p=0.006)	−0.70 (p=0.000)
FI (%)	−0.001 (p=0.996)	−0.30 (p=0.118)	0.01 (p=0.978)	0.08 (p=0.663)	0.20 (p=0.309)

TT= total time; PT= peak time; FI= fatigue index; (T30) = 30 m straight sprint test; CMJ= countermovement jump, 5JT= five jump test; AnSR= anaerobic speed reserve; [LA (3′)] = lactate concentration after RSA test.

**Table 3:** Stepwise multiple regression analyses for the repeated sprint test indices

	Criteria tests	R	R^2^	SE	P
TT (s)	AnSR (km·h^−1^)	0.68	0.47	1.90	0.000
PT (s)	AnSR (km·h^−1^)	0.71	0.50	0.18	0.000

R = multiple-correlation coefficient; R² = shared variance; SE = standard error of prediction; TT= total time; PT= peak time.
